# Social Return on Investment (SROI) of mental health related interventions—A scoping review

**DOI:** 10.3389/fpubh.2022.965148

**Published:** 2022-12-09

**Authors:** Rajendra Kadel, Anna Stielke, Kathryn Ashton, Rebecca Masters, Mariana Dyakova

**Affiliations:** WHO Collaborating Centre on Investment for Health and Wellbeing, Public Health Wales NHS Trust, Cardiff, United Kingdom

**Keywords:** review, SROI, interventions, mental health and wellbeing, social value

## Abstract

**Background:**

There is a growing recognition of the need to effectively assess the social value of public health interventions through a wider, comprehensive approach, capturing their social, economic and environmental benefits, outcomes and impacts. Social Return on Investment (SROI) is a methodological approach which incorporates all three aspects for evaluating interventions. Mental health problems are one of the leading causes of ill health and disability worldwide. This study aims to map existing evidence on the social value of mental health interventions that uses the SROI methodology.

**Methods:**

A scoping evidence search was conducted on Medline, PubMed, Google Scholar and relevant gray literature, published in English between January 2000 and March 2021 to identify studies which capture the SROI of mental health interventions in high- and middle-income countries. Studies that reported mental health outcomes and an SROI ratio were included in this review. The quality of included studies was assessed using Krlev's 12-item quality assessment framework.

**Results:**

The search identified a total of 435 records; and 42 of them with varying quality met the study inclusion criteria. Most of the included studies (93%) were non-peer reviewed publicly available reports, predominantly conducted in the United Kingdom (88%); and majority (60%) of those studies were funded by charity/non-for-profit organizations. Out of 42 included studies, 22 were targeted toward individuals experiencing mental health problems and the remainder 20 were targeted to vulnerable groups or the general population to prevent, or reduce the risk of poor mental health. Eighty-one percent of included studies were graded as high quality studies based on Krlev's 12-item quality assessment framework. The reported SROI ratios of the included studies ranged from £0.79 to £28.00 for every pound invested.

**Conclusion:**

This scoping review is a first of its kind to focus on SROI of mental health interventions, finding a good number of SROI studies that show a positive return on investment of the identified interventions. This review illustrates that SROI could be a useful tool and source of evidence to help inform policy and funding decisions for investment in mental health and wellbeing, as it accounts for the wider social, economic and environmental benefits of public health interventions. More SROI research in the area of public health is needed to expand the evidence base and develop further the methodology.

## Introduction

Mental health problems (MHPs) are one of the leading causes of ill health and disability worldwide (1, 2). One in four people experience mental health problems at some point in their lives, and many of them go undiagnosed (3). MHPs are major contributors to the global burden of disease, with the share of about 14% of years lived with disability (YLDs) and 4.9% of disability adjusted life years (DALYs) in 2018 (4). There is huge imbalance between health burden, financing and service delivery in mental health in several countries with different income levels (5).

MHPs cause major economic consequences in terms of treatment, productivity and welfare/benefits to individuals, families and wider society (6). It is estimated that MHPs will cost $16 trillion US dollars (equivalent to £11 trillion, price year 2010) to the global economy over 20 years by 2030 (7). It is also estimated that MHPs cost the UK economy between £70–100 billion a year, about 4.5% of gross domestic product (8). The latest estimate by Deloitte showed that MHPs cost the UK employers between £33–42 billion a year (9).

Several interventions have been conducted to improve mental health and wellbeing across the life course (10). The economic evaluations of such interventions have also been well studied to see whether these interventions are financially worth-investing (11–14). However, these evaluations have not sufficiently captured the wider social value and impact of the interventions. One of the common evaluation tools to capture the wider outcomes, impact and related social value could be the Social Return on Investment (SROI) analysis (15).

SROI is an analysis framework to identify, measure and report the social, economic and environmental benefits generated from the interventions (15). The analysis is based on the concept of the theory of change and logic model. The foundations of SROI analysis is based on the traditional economic evaluations (16), and the value generated by the programme is relied upon the strong engagement of different levels of stakeholders who are directly or indirectly impacted by the programme (15, 17). A detailed SROI analysis process has been described elsewhere (15, 18). In brief, there are six stages of carrying out an SROI analysis. The first stage is establishing scope and identifying key stakeholders. In this stage, clear boundaries of what the analysis will cover, and who will be involved in the process and at what capacity. The second stage is related to mapping outcomes. In this stage, we will develop an impact map with the involvement of stakeholders, and the impact map should clearly visualize the relationship among inputs, outputs and outcomes. The third stage is evidencing outcomes and giving them a value. This stage involves exploring data to demonstrate whether the programme yields outcomes and then valuing them in a monetary term. In the fourth stage, the impact of the programme is established based on collected information and adjusted for other factors that could influence the overall results of the programme. The fifth stage calculates the SROI ratio by adding up all the benefits or savings and dividing it by the total investment in the programme, and performing sensitivity analysis. The final stage of the SROI analysis is related to reporting, using and embedding which involves sharing SROI findings with wider stakeholders, responding to their queries and embedding good practice and verification of the report.

Previous reviews on SROI included mental health interventions along with other public health interventions (18, 19), but to our knowledge, this scoping review is the first in its kind to exclusively focus on the SROI of mental health related interventions. The aim of this scoping review is to explore and map existing evidence on the social value of (public) mental health interventions that use the SROI method. The objectives of this review are to: (a) identify general characteristics of the SROI studies; (b) outline the reported SROI values; (c) assess methodological quality of SROI evidence; and (d) identify gaps in current literature related to the social value of mental health interventions. The findings can inform policy makers, budget holders and funding agencies about the value of investing in mental health and wellbeing to generate wider social, economic and environmental returns toward building healthier populations, communities and the planet.

## Methods

This review is limited to studies which illustrate the SROI of public health interventions to improve mental health and wellbeing. The interventions could be targeted to people at any age group who were at risk of, or currently experiencing mental health problems.

### Search process

PubMed/Medline, Google Scholar and relevant gray literature were searched for published records between January 2000 and March 2021. The search strategy combined the terms related to mental health and wellbeing, and Social Return on Investment. Potential relevant studies were first screened based on titles and abstracts, and the full texts were then retrieved for those likely to meet the inclusion criteria. The screened studies were independently assessed by two authors for inclusion in the review.

### Study inclusion criteria

This scoping review was restricted to publication in English and included both scientific and gray literature of primary studies published between January 2000 and March 2021. Studies with any study design that reported SROI of interventions related to mental health and wellbeing, conducted in high and middle income countries were included.

### Data extraction

Data was extracted from the eligible studies on population, intervention, outcomes and economic results in an independently developed data extraction form. Major economic findings of the SROI analysis and comprehensive data on total investment and realized benefits of the mental health interventions, or interventions targeted to improve mental health and wellbeing were extracted. Economic results were shown in monetary value of the return on every pound/dollar invested in the intervention.

### Methodological quality assessment

A 12-point quality assessment framework developed by Krlev et al. (20) was used to assess the methodological quality of SROI studies. This quality assessment tool was used in previous reviews of the SROI studies (18, 19, 21). The quality assessment framework has proposed five quality dimensions spread over 12 different quality criteria. The quality assessment results of the included studies are presented in Table 1.

**Table 1:** SROI findings.

**Authors**	**SROI type**	**Sample size**	**Intervention**	**(Mental health) Outcomes**	**Time horizon**	**Costs**	**SROI ratio**	**Price year**	**Sensitivity analysis**	**Quality grade**
**A) Interventions targeted to people experiencing mental health problems**										
Robinson (22)	Forecast	10	Artist for mental health mindfulness project	Improved mental health awareness, Develop skills on mindfulness, friendship, and sense of belongings	60 months	Investment = £685 Benefits = £4435	SROI = £6.48/£1 invested	2020 (£)	Yes	High
Lakhotia (23)	Forecast	65	Incredible years parenting programme	Reduced child conduct problems, Family wellbeing, Reduced social and fiscal costs	36 months	Investment =$484,196 Benefits = $1,815,855	SROI = $3.75/$1 invested	2017 (AUD$)	Yes	High
Envoy Part-nerships (24)	Evaluative	569	Multilingual emotional wellbeing support service	Reduced anxiety and depression, Improved mental wellbeing Improved resilience and coping	36 months	Investment = £146,200 Benefits = £702,000	SROI = £3.20/£1 invested	2018 (£)	No	Low
Lloyd (25)	Forecast	153	Peer mentor service	Improved mental health, Improved family relationships, Felt less alone and isolated	12 months	Investment = £273,047 Benefits = £1,854,760	SROI = £6.79/£1 invested	2017 (£)	Yes	High
McCorriston (26)	Evaluative	153	Peer education programme	Improved mental health and wellbeing, Improved family relationship, Less visit to mental health service	24 months	Investment = £11,151 Benefits = £314,483	SROI = £28/£1 invested	2017 (£)	Yes	High
Dayson (27)	Evaluative	246	Social prescribing	Improved mental health and wellbeing, Improved relations with family and friends, Employment opportunities	24 months	Investment = £349,300 Benefits = £309,795	SROI = £0.79/£1 invested	2015 (£)	No	Low
Richardson (28)	Forecast	4,482	Future digital inclusion	Improved health and wellbeing, Better quality relationships, Reduced social isolation	24 months	Investment = £3,500,000 Benefits = £15,000,000	SROI = £4.28/£1 invested	2014 (£)	Yes	High
Whelan (29)	Evaluative	70	Creative alternatives arts	Improved mental wellbeing, Reduced GP visits, Increased social activities	12 months	Investment = £40,000 Benefits = £165,000	SROI = £4.12/£1 invested	2015 (£)	Yes	High
Marsh (30)	Forecast	3,271	Local area coordination	Reduced anxiety and depression, Improved mental wellbeing, Increased self-confidence	36 months	Investment = £1,759,445 Benefits = £6,468,246	SROI = £3.68/£1 invested	2016 (£)	No	Low
Biggs (31)	Evaluative	89	The Art-Ease project	Reduced anxiety and depression, Increased confidence and self-worth, Reduced drugs and alcohol problems	19 months	Investment = £35,586 Benefits = £202,952	SROI = £3.31/£1 invested	2014 (£)	No	Low
Weld (32)	Evaluative	79	Healthy connection project	Improved mental wellbeing, Reduced suicidal rates, Reduced social isolation	15 months	Investment = £48,820 Benefits = £181,894	SROI = £3.73/£1 invested	2013 (£)	Yes	High
Shipway (33)	Evaluative	660	Creative arts	Reduced anxiety and depression, Improved mental health, Increased confidence	18 months	Investment = £489,000 Benefits = £2,497,000	SROI = £5/£1 invested	2013 (£)	No	Low
Arvidson (34)	Forecast	39	Community befriending programme	Improved mental health, Reduced behavioral problems,	36-−360 months	Investment = No reported Benefits = Not reported	SROI = £3/£1 invested (3 years) SROI = 6.50/£1 invested (30 years)	Not reported	No	High
Quality Matters (35)	Evaluative	36	Mojo, creating male space	Improved mental health and wellbeing, Reduced self-harming behavior, Improved relations with family	13 months	Investment = €111,293 Benefits = €477,246	SROI = €4.26/€1 invested	2012 (€)	Yes	High
Goodspeed (36)	Evaluative	1,136	Substance misuse service	Improved mental health, Reduced substance use, Improved relations with family	12 months	Investment = £3,368,809 Benefits = £29,925,400	SROI = £8/£1 invested	2013 (3)	Yes	High
NEF Consul-ting (37)	Evaluative	293	Sustainable commissioning model	Improved mental health and wellbeing, Increased social networks	12 months	Investment = £689,515 Benefits = £4,700,000	SROI = £5.75/£1 invested	2009 (£)	Yes	High
Szplit (38)	Forecast	45	Individual placement and support	Improved mental wellbeing, Improved relations with family, Increased confidence	12 months	Investment = £77,822 Benefits = £526,885	SROI = £5.77/£1 invested	2010 (£)	Yes	High
Ireland (39)	Evaluative	21	Gardening in Mind	Improved mental health Strong family and social ties	12 months	Investment = £57,906 Benefits = £117,961	SROI = £2.04/£1 invested	2009 (£)	Yes	High
Leck (40)	Evaluative	83	The Houghton project	Improved mental health, Become more confident Feel more positive	12 months	Investment = £154,386 Benefits = £677,207	SROI = £4.39/£1 invested	2010 (£)	Yes	High
GAMP (41)	Forecast	160	Scotia clubhouse programme	Improved mental health, Wider social network, Better quality relationships	60 months	Investment = £301,197 Benefits = £1,621,891	SROI = £5.38/£1 invested	2010 (£)	Yes	High
Carrick (42)	Evaluative	104	Health walks programme	Reduced need of counseling service, Reduced need of medical prescription, Less need of hospital stays	36 months	Investment = £84,500 Benefits = £969,591	SROI = £11.47/£1 invested	2004 (£)	Yes	High
University of Worcester (43)	Forecast	16	Nineveh ridge care farm	Improved mental wellbeing, Reduced drugs and alcohol dependence, Improved confidence and quality of life	12-−24 months	Investment = £60,500 Benefits = £205,167	SROI = £2.40/£1 invested	2011 (£)	Yes	High
Goodspeed (44)	Forecast	105	Workwise activities	Improved mental wellbeing, Increased confidence	12 months	Investment = £490,456 Benefits = £1,494,484	SROI = £3/£1 invested	2009 (£)	Yes	High
Somers (45)	Evaluative	32	MillRace IT project	Improved mental health	12 months	Investment = £10,325 Benefits = £87,150	SROI = £7.44/£1 invested	2005 (£0	Yes	High
**B) Interventions targeted to vulnerable groups for mental health problems**										
Isard (46)	Evaluative	16	DIAL House	Increased mental wellbeing, Increased quality of family relationships, Decreased drugs and/or alcohol use	24 months	Investment = €283,986 Benefits = €1,633,718	SROI = €5.75/€1 invested	Not reported	Yes	High
Tokarova (47)	Evaluative	22	Works' wellbeing programme	Improved wellbeing and mental health, Increased societal relationships	36 months	Investment = £11,300 Benefits = £42,270	SROI = £3.74/£1 invested	2012 (£)	Yes	High
RM Insight (48)	Evaluative	77	Family action mental health project	More resilient mental health, Improved confidence and network	12 months	Investment = £40,000 Benefits = £78,000	SROI = £1.94/£1 invested	2011 (£)	Yes	High
Deslandes (49)	Forecast	569	Community arts in mental health	Improved mental health and wellbeing, Improved confidence	36 months	Investment = £16,420 Benefits = £153,940	SROI = £9.38/£1 invested	2010 (£)	Yes	High
Cawley (50)	Forecast	55	Changing mind programme	Increased mental wellbeing, Increased confidence, Reduced visits to healthcare	60 months	Investment = £74,047 Benefits = 540,413	SROI = £8.78/£1 invested	2009 (£)	Yes	High
**C) Interventions targeted to promote mental health and wellbeing among general population**										
Bagnall (51)	Evaluative	77	Nature conservation activities	Improved wellbeing scores, Increased level of nature relatedness	12 months	Targeted project Investment = £98,654 Benefits = £1,162,607 Volunteering project Investment = £31,584 Benefits = £459,453	Targeted project SROI = 11.78/£1 invested Volunteering SROI = £14.55/£1 invested	Not reported	Yes	Low
Lloyd (52)	Evaluative	120	Arfon social prescribing model	Improved mental health, Reduced loneliness and isolation, Reduced demand on GP visits	18 months	Investment = £71,992 Benefits = £246,123	SROI = £3.42/£1 invested	2017 (£)	Yes	High
Envoy part-nerships (53)	Forecast	33	Selfcare social prescribing	Reduced depression and anxiety Reduced need for hospitalisations	24 months	Investment = £250,000 Benefits = £470,025	SROI = £6.25/£1 invested	2017 (£)	Yes	High
Envoy part-nerships (54)	Evaluative	300	Community champions programme	Improved wellbeing, Improved community cohesion, Resources savings to healthcare	24 months	Investment = £930,000 Benefits = £5,000,000	SROI = £5/£1 invested	2016 (£)	Yes	High
Hackett (55)	Evaluative	75	Residential treatment programme	Reduced mental health admissions, Reduced substance use admissions	72 months	Investment = £894,965 Benefits = £7,273,226	SROI = £7/£1 invested	2010 (CAD$)	Yes	High
Lloyd (56)	Evaluative	172	Carers outreach programme	Improved mental health, Increased confidence, Feeling less alone	12 months	Investment = £26,215 Benefits = £152,629	SROI = £5.82/£1 invested	2016 (£)	Yes	High
Bertotti (57)	Forecast	30	Highway house—homeless shelter project	Improved mental health, Reduced healthcare expenses	60 months	Investment = £94,910 Benefits = £537,761	SROI = £5.67/£1 invested	2014 (£)	Yes	High
Warby (58)	Evaluative	200 HH	Community health champions	Improved mental wellbeing, Sense of community and cohesion	36 months	Investment = £90,000 Benefits = £322,000	SROI = £3.85/£1 invested	2014 (£)	No	Low
Whelan (59)	Evaluative	307	Taiko drumming for health	Improved mental health and wellbeing, Improved social values	12 months	Investment = £15,965 Benefits = £120,938	SROI = £8.58/£1 invested	2012 (£)	Yes	High
Wright (60)	Evaluative	832	Building social capital project	Increased resilience and self-esteem, Increased positive functioning, Supportive relationships	12 months	Investment = £338,718 Benefits = £929,790	SROI = £2.75/£1 invested	2010 (£)	Yes	High
Goodspeed (61)	Evaluative	73	Family intervention project	Parents felt less anxious and depressed, Improved family and life relationships, Improved behaviors	18 months	Investment = £304,108 Benefits = £1,300,402	SROI = £4/£1 invested	Not reported	Yes	High
Visram (62)	Evaluative	3,179	Integrated health and wellbeing services	Health gain from emotional wellbeing	18 months	Investment = £3,528,894 Benefits = £9,756,450	SROI = £3.45/£1 invested	2014 (£)	No	High
McGrath (63)	Evaluative	78	Circus arts training	Improved mental wellbeing, Improved confidence, Improved socialization skills	30 months	Investment = $550 Benefits = $3,685	SROI = $7/$1 invested	2016 (AUD$)	No	High

One point is given to each criterion, if it is present and zero points otherwise. A 70% benchmarking as proposed by Krlev and colleagues in 2013 was used as a “good score” to rate the study as “high quality” and “low quality” if the study scored < 70% (20).

## Results

The preferred Reporting Items for Systematic Review and Meta-analyses (PRISMA) guideline (64) was followed to report the findings of the scoping review.

**Figure 1:**
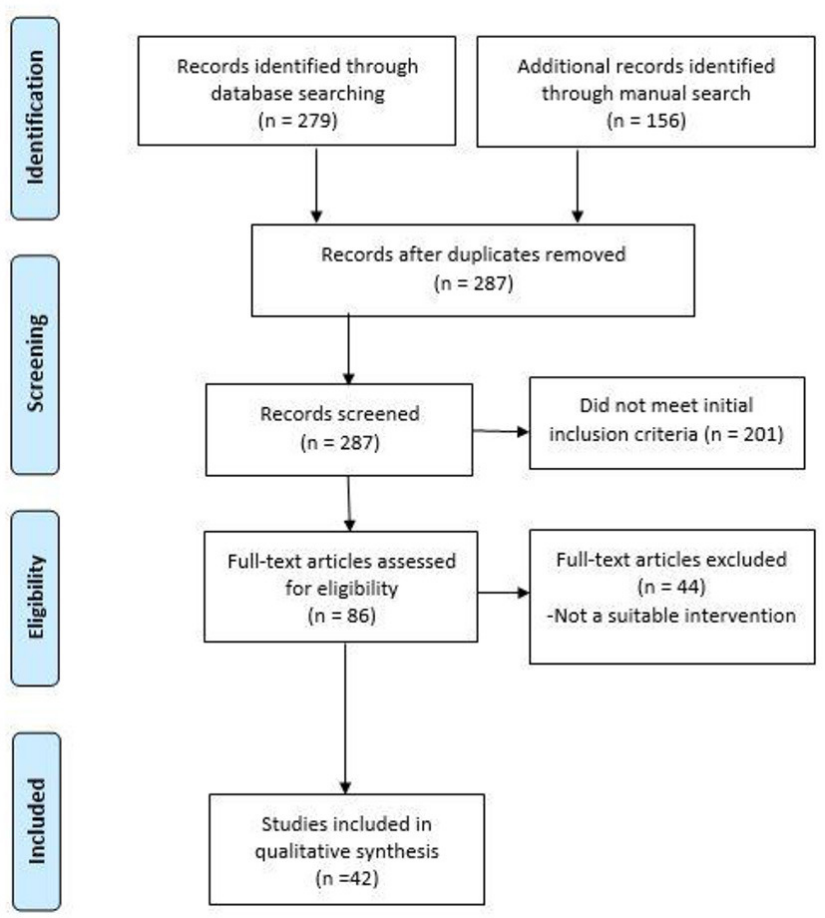
PRISMA flow chart.

The search hit a total of 435 records, 279 were from database searches and 156 from manual search (Figure 1). In total, 287 records were included for initial screening after duplicates were removed. Two hundred and one records were excluded from the initial title and abstract screening, leading to 86 records for full-text review and at this stage 44 records did not meet the inclusion criteria, which yielded 42 studies for inclusion in the final review.

Table 2 summarizes the study characteristics; and Table 1 summarizes the SROI findings.

**Table 2:** Study characteristics.

**Authors**	**Year**	**Country**	**Commissioned by**	**Studied by**	**Target population**	**Stakeholders**	**Settings**	**Publication type**	**Study assurance**
Robinson (22)	2020	England, UK	Non-profit organization	Consultancy service	Affected population—young adults	Young adults, volunteers, family members, staff	Community	Report	Yes
Lakhotia (23)	2019	New Zealand	Non-profit organization	Consultancy service	Affected population—Children	Parents and caregivers, children, staff	Community	Report	Yes
Envoy Part-nership (24)	2019	England, UK	Public agency—Local Borough	Consultancy service	Affected population—BAME	Service users, family members/carers, NHS, Local authorities	Residential	Report	No
Lloyd (25)	2018	Wales, UK	Public agency—NHS Wales	Non-profit organization	Affected population—Veterans	Veterans, family members, peer mentors, NHS Wales,	Community	Report	Yes
McCorriston (26)	2018	England, UK	Public agency -NHS England	Academic Institution	Affected population—Adults	Family/friends and loved ones, peer support workers, Foundation trust, NHS/state	Community	Report	No
Dayson (27)	2017	England, UK	Public agency -NHS England	Academic Institution	Affected population—Adults	Service users, carers, family and friends, local organizations, NHS	Community	Report	No
Richardson (28)	2016	England, UK	Charitable organization	Non-profit organization	Affected population—Working age	Jobseekers, low income groups, disabled people, SMEs, NHS	Community	Report	No
Whelan (29)	2016	England, UK	Non-profit organization	Academic Institution	Affected population—Adults	Creative alternatives attendees, NHS	School	Report	No
Marsh (30)	2016	England, UK	Public Agency—Local Borough	Consultancy service	Affected population—Adults	Service users, family members and neighbors, local area coordinators, NHS, local authority, foundation trust	Community	Report	Yes
Biggs (31)	2015	England, UK	Charitable organization	Academic Institution	Affected population—Adults	Service users, project staff, NHS, local authority	Community	Report	No
Weld (32)	2015	England, UK	Non-profit organization	Academic Institution	Affected population -Adults	Project participants, project staff and volunteers, NHS, local authority	Community	Report	No
Shipway (33)	2015	England, UK	Social Enterprise	Consultancy service	Affected population—Children and Adults	Service users, members, staff and volunteers, carers and families, NHS	Community	Report	No
Arvidson (34)	2014	England, UK	Charitable organization	Academic Institution	Affected population—Mothers	Service users, volunteers, NHS	Community	Article	No
Quality Matters (35)	2014	Ireland	Non-profit organization	Non-profit organization	Affected population—Adults	Project participants, family members, professionals, referral agents, Health Service Executive	Community	Report	Yes
Goodspeed (36)	2014	England, UK	Public Agency—NHS England	Non-profit organization	Affected population—Adults	Service users, families, communities, local authority, project staff, NHS, housing providers	Community	Report	Yes
NEF Consul-ting (37)	2013	England, UK	Public Agency—Local Borough	Consultancy service	Affected population—Adults	Service users, volunteers, community, local authority	Community	Report	No
Szplit (38)	2013	Wales, UK	Public Agency—DWP	Non-profit organization	Affected population—Adults	Clients, families, staff, employers, NHS and State	Community	Report	Yes
Ireland (39)	2013	England, UK	Charitable organization	Non-profit organization	Affected population—Adults	Service users, family carers, NHS, funders	Residential	Report	Yes
Leck (40)	2012	England, UK	Non-profit organization	Academic Institution	Affected population-−14 years and above	Service users, family members, NHS, volunteers, employees, host farmers	Community	Report	No
GAMP (41)	2011	Scotland, UK	Charitable organization	Non-profit organization	Affected population -Adults	Clubhouse members, local authority, health board, referring agencies,	Community	Report	Yes
Carrick (42)	2011	Scotland, UK	Public Agency—Local Borough	Charity	Affected population—Older people	Walkers, walk leaders, volunteers, NHS, local authority, staff	Community	Report	No
University of Worcester (43)	2011	England, UK	Non-profit organization	Academic Institution	Affected population—Children & Adults	Service users, family members, school children, farmers, volunteers, placement commissioners, government	Community and School	Report	Yes
Goodspeed (44)	2009	England, UK	Non-profit organization	Consultancy service	Affected population—Adults	Trainees, family and friends, employees, volunteers, local authority, NHS,	Workplace	Report	Yes
Somers (45)	2006	England, UK	Non-profit organization	Consultancy service	Affected population—Adults	Participants, family members, employees, local partner, local authority, NHS	Workplace	Report	No
Isard (46)	2020	Ireland	Non-profit organization	Non-profit organization	Vulnerable population—Young adults	Young adults, family members, service providers, local authority	Residential	Report	Yes
Tokarova (47)	2014	England, UK	Charitable organization	Academic Institution	Vulnerable population—Adults (carers)	Project participants, funders, NHS, local authority	Community	Report	No
RM Insight (48)	2012	England, UK	Charitable organization	Consultancy service	Vulnerable population—children and adults	Adult and child participants, family members, volunteers, school, NHS	Community	Report	Yes
Deslandes (49)	2011	England, UK	Charitable organization	Consultancy service	Vulnerable population—Adults (artists)	Project participants, community, NHS	Community	Report	Yes
Cawley (50)	2011	England, UK	Public Agency—NHS England	Academic Institution	Vulnerable population—Adults	Trainees, training providers, NHS, local authority	Community	Report	No
Visram (62)	2020	England, UK	Public Agency—Local Borough	Academic Institution	General population—Adults	project participants, NHS, public sectors	Community	Article	No
McGrath (63)	2019	Australia	Academic institution	Academic Institution	General population—Children	Trainees, parents, trainers, staff	School	Article	No
Bagnall (51)	2019	England, UK	Charitable organization	Academic Institution	General population—Adults	Project participants, volunteers, staff,	National	Report	No
Lloyd (52)	2018	Wales, UK	Charitable organization	Consultancy service	General population—Adults	Service users, family members, charity, NHS	Community	Report	Yes
Envoy Part-nership (53)	2018	England, UK	Public Agency—Local Borough	Consultancy service	General population—Elderly	Patients, family members, NHS, local authority	Community	Report	No
Envoy Part-nership (54)	2018	England, UK	Public Agency—Local Borough	Consultancy service	General population—Adults	Champions, residents, children, local authority, state	Community	Report	No
Hackett (55)	2017	Canada	Charitable organization	Academic Institution	General population -Teenagers	Participants, parents/guardians, health systems, state (labor force)	School	Report	No
Lloyd (56)	2016	Wales, UK	Charitable organization	Consultancy service	General population—Parents	Parents, children, NHS, carers outreach, child service provider,	Community	Report	No
Bertotti (57)	2015	England, UK	Charitable organization	Academic Institution	General population—Homeless people	service users, service provider, local authority, NHS, state	Residential	Report	No
Warby (58)	2014	England, UK	Charitable organization	Consultancy service	General population—Women and Children	Champions, residents, children, local authority, state	Residential	Report	No
Whelan (59)	2013	England, UK	Public Agency—Local Borough	Academic Institution	General population—Children and adults	Drummers, volunteers, project management team	School and day center	Report	No
Wright (60)	2012	England, UK	Public Agency—Local Borough	Consultancy service	General population—Adults	Service users, family members and carers, volunteers	Community	Report	No
Goodspeed (61)	2010	England, UK	Charitable organization	Consultancy service	General population—Parents and children	Children and young people, family and carers, police, NHS, local authorities	Community	Report	Yes

### Study characteristics

Most of the included studies (93%) were non-peer reviewed publicly available reports, predominantly conducted in the UK (88%). The majority (60%) of the studies were funded by either charity or non-for-profit organizations, while 36% from NHS and local government agencies. We also found that the majority (74%) of the studies were conducted by either private consultancy firms or academia. Except two (44, 45), all other studies were conducted 2010 onwards.

More than two-third of the studies were conducted at the community level. In 57% of the studies, the direct beneficiaries were people experiencing some form of existing mental health problems. Majority of the studies in the review included direct beneficiaries from specific age groups (children, teenagers, youth, adults, working age, elderly). Some studies included specific population groups such as veterans, Black and Ethnic Minorities, mothers, carers, artists, parents, and homeless people. In addition to service users or direct beneficiaries, the studies included different stakeholders, such as volunteers, family members/friends, service providers, schools, local authorities, local organizations, NHS/health systems, other public services, referral agencies, charities, commissioners/funding agencies, national government. These SROI studies ranged in sample size from as low as 10 (22) to as high as 4,482 (28).

The studies evaluated SROI of different interventions related to mental health and wellbeing, including: arts for mental health (22, 29, 31, 33, 49, 63); workplace intervention (38, 44, 45, 47); farm or gardening activities (39, 40, 43); social prescribing (27, 52, 53); peer support (25, 26, 41); family support (23, 48, 61); residential interventions (46, 55, 57); awareness/training (32, 42, 50); community health champions (54, 58); ethnicity or culture—focused activities (24, 59); treatment/therapy (36, 62); digital inclusion (28); creating male space (35); nature conservation activities (51) and other community level activities (30, 34, 37, 56, 60).

Quality assessment: Out of 42 studies, 81% of the studies were considered as being high quality studies (Table 1). It was found that about 40% of the studies were submitted to and approved by the Social Value International for assurance (Table 2). Furthermore, we also found that 79% of the studies conducted sensitivity analyses to provide robustness of the SROI results (Table 1).

### SROI results

In two-thirds of the studies, the SROI analyses were evaluative, with the remainder being forecast analyses. The evaluation time frame ranged from 1–6 years, with an exception of up to 30 years which evaluated the SROI of a community befriending programme to prevent post-natal depression (34).

Though there was wide variation in methodological quality and intervention types, most studies clearly illustrate the positive SROI of the interventions aimed at reducing mental health problems, and/or improving mental health and wellbeing. There is significant variation of the SROI ratio between studies—ranging from £0.79 (27) to £28 (26) for every pound invested. The SROI findings are further categorized on the basis of the mental health status/risk of the target population of the included studies. The SROI ratios of the interventions which were targeted to people who were experiencing mental health problems ranged from £0.79 to 28 for every £1 invested in the intervention. The interventions which were targeted to vulnerable/risky populations for mental health problems showed the SROI ratios that were ranged from £1.94 to 9.38 for every £1 invested. Similarly, the interventions to promote mental health and wellbeing of the general populations showed the SROI ratios that were ranged from £2.75 to 14.55 for every £1 invested in the intervention.

Twelve months was the lowest analysis time horizon where family action mental health project (48) yielded the lowest SROI of £1.94 for every pound invested, and the nature conservation activities of Wildlife Trust (51) yielded the highest SROI ratio of £14.55 for every pound invested. Thirty-year was the highest/longest SROI forecast analysis time horizon where an SROI of a community befriending programme to the families affected by post-natal depression (34) was estimated with a benefit of £6.50 for every pound invested. The detailed SROI findings of a review is presented in Table 1.

## Discussion

This scoping review aims to explore the application of the SROI method to evaluate (public) mental health interventions. Compared to previous reviews on the SROI of public health interventions, which also included studies on the SROI of mental health interventions (18, 19), our scoping review incorporates studies with mental health intervention or studies that included mental health and/or wellbeing outcomes while evaluating social value of the intervention. The application of SROI to evaluate the wider social benefits of the mental health interventions could be used to inform policy decisions and investment prioritization in mental and wider public health.

Our review has found a good number of published reports that have shown a sizeable SROI of interventions addressing/preventing mental health issues or improving mental health and wellbeing. Overall, 42 studies with varying methodological quality were included in this review. The SROI ratios of the included studies ranged from £0.79 to 28 for every pound invested. Eighty-one percent of the studies were identified as high quality, using the Krlev's 12-item quality assessment framework (20), which allows studies for comparisons with relevant previous SROI reviews (18, 19). Our review findings are consistent with previous review findings related to SORI of mental health interventions.

The SROI method is being increasingly used to evaluate the wider impact and social value of various enterprises (65) as well as different programmes in health sectors (18) for the past two decades. The evaluation of public health interventions using a Social Value approach and SROI method have rapidly increased after 2010 in the health sector, especially by UK public and not-for-profit organizations. This might be due to the development of the guideline to SROI in 2009 (66) and the subsequent endorsement of the Public Service (Social Value) Act 2012 (67). However, there is little interest or motivation from evaluators and researchers to publish such evidence in peer-reviewed journals. This may partly be due to the introduction of SROI to evaluate the social value of the programmes delivered through not-for-profit or third sector organizations where publishing findings in academic journals may not be the priority; or partly due to potential “methodological fallacy” of the SROI approach perceived by the academic scholars.

The study interventions identified in this review were targeted either to reduce or prevent mental health problems, or to promote mental health and wellbeing, but interestingly none of the included SROI studies evaluated clinical treatment of mental health problems. We also found that studies vary widely in terms of types of interventions used, ranging from creative arts to nature conservation activities. The review showed considerable variation in the SROI findings according to mental health risk status of the target population in the included studies, but none of the studies showed negative SROI results. This implies that interventions that aimed to reduce/prevent mental health problems or promote mental health and wellbeing could have potential to yield positive Social Return on Investment.

This review also highlights the relevance of the SROI method to improve the measurement, valuation and reporting of the influence of mental health and wellbeing related intervention(s) to the wider society, economy and the planet, compared to traditional economic evaluations (18).

There is growing interest and drive from government and non-profit organizations to assess and maximize the value for money, and social value, of the public health interventions (62). Our review shows good SROI values of public health interventions for the prevention or reduction of mental health problems and promotion of mental health and wellbeing. These findings provide substantial evidence and a helpful insight related to a number of mental health interventions, to support policy makers and budget holders when taking decisions, evaluating programmes and prioritizing funding and investment in mental and wider public health and wellbeing.

## Study limitations

Our review has several limitations. Only English language studies were included, while there might be studies conducted in other languages. We only included published SROI studies; there could be some studies which have not been published in the databases and sources searched. The existing Krlev's 12-item quality assessment framework has not been updated; some of the quality criteria have been subjective and difficult to judge, which may affect the reliability of the study results. We, however, used the quality criteria to the best of our ability to consistently apply throughout the included studies. There has been a high variability in the way the included studies have been conducted, which has limited the capacity to collate or draw summary/collective findings in the review. Due to large heterogeneity in sample size, intervention methods and benefit periods of the SROI ratios, it has not been possible to quantitatively synthesize the SROI results.

## Gaps for further research

Our review has aimed to explore the existing evidence on SROI of mental health related interventions, but has not assessed other existing methods that might be also used to assess the value of mental health related interventions. Further research is needed to understand whether other existing methods could provide robust evidence in terms of identifying, measuring and reporting of the wider benefits/outcomes, impact and social value of interventions related to mental health and wellbeing. Current focus of the SROI data collection process is based on input/output of the intervention, but it is necessary to focus more on impact-oriented measures to capture their true value in the mid/long-term. There is also a need to publish more studies from SROI research work in the academic (peer reviewed) journals to attract wider academic audiences to explore and develop further this method and its application venues. More SROI research in the area of public health is needed to expand the evidence base and better inform investment prioritization, commissioning/funding decisions and programme improvement.

## Author contributions

RK designed a scoping review, developed search strategies, assessed studies for inclusion, analysis, and drafting an initial manuscript. AS involved in the assessment of studies for inclusion. RK, AS, KA, RM, and MD subsequently revised and approved the final manuscript. All authors contributed to the article and approved the submitted version.
